# Characterisation of *Yersinia pseudotuberculosis* bioserotype 1/O:2b ST16 causing a bovine mastitis infection in a dairy cow in Germany

**DOI:** 10.3389/fvets.2026.1833805

**Published:** 2026-06-03

**Authors:** Sonja Bäßler, Catharina Pölzelbauer, Claudia Jäckel, Stefan Hertwig, Jens A. Hammerl

**Affiliations:** 1 Baden Württemberg Udder Health Service, Tierseuchenkasse Baden-Württemberg, Fellbach, Germany; 2 Bacteriology Laboratory, Chemical and Veterinary Analysis Agency Stuttgart (CVUAS), Fellbach, Germany; 3 Consiliary Laboratory for Yersinia in Food, Department Biological Safety, German Federal Institute for Risk Assessment (BfR), Berlin, Germany

**Keywords:** diversity, genomics, livestock, mastitis, milk-borne diseases, virulome, *Yersinia pseudotuberculosis*

## Abstract

**Introduction:**

The zoonotic bacterium *Yersinia pseudotuberculosis* (YPS) primarily causes gastrointestinal and extraintestinal diseases in animals, but can also infect humans. Due to the concerted action of a broad spectrum of virulence determinants associated with an infection by YPS, the host specificity of certain phylotypes and determinants affecting the epidemiology of the disease are still poorly understood. In 2025, a case of mastitis of a cow in Germany was attributed to YPS, with the bacterium being isolated at various time points.

**Methods:**

The isolates were analysed in-depth using classical and whole-genome-based approaches to reveal their phenotypic and genetic properties.

**Results:**

Mastitis was caused by a single strain (25-YE00352) of the bio/serotype 1/O:2, which belongs to multilocus-sequence type ST16 (phylotype). Except for the *Yersinia* adhesion pathogenicity island YAPI, the genome of 25-YE00352 lacked other main virulence factors, including the pYV virulence plasmid, the YPS-derived mitogen superantigen, and the pathogenicity island HPI. Phylogenetically, 25-YE00352 resembles isolates recently described from wildlife and captive animals in Germany.

**Conclusion:**

To the best of our knowledge, this is the first report from Germany on a YPS isolate recovered from a chronic case of mastitis in a cow.

## 1 Introduction

*Yersinia pseudotuberculosis* (YPS) is a species that naturally resides in the gastrointestinal tract of pets, livestock and wildlife and can provoke severe human infections (1–6). Zoonotic infections usually occur occasionally, resulting from a faecal-oral transmission of bacteria from animals to humans. YPS can also cause human infections through the consumption of contaminated food such as milk products, undercooked or raw meat, fresh vegetables, vegetable juices and sandwiches (7). Due to its ability to grow at low temperatures (0–4 °C), the bacterium can multiply up to infectious doses in food stored at refrigerated temperatures (8). Large outbreaks in humans have especially been reported in countries with cold climates (1, 9, 10) e.g., Finland in 2018, where raw milk products were identified as the source of an outbreak (9).

In warm-blooded animals, particularly rodents and birds, YPS can cause tuberculosis-like fatal diseases (11–13). As the symptoms of the disease are rather non-specific, infection of animals usually goes unrecognised, and YPS is often only detected post-mortem through the recovery of bacteria from abscesses in the liver and spleen (6, 14, 15). The susceptibility of animals to YPS generally depends on the health and genetic status of the host, as well as prevailing stress conditions (16, 17). Besides common clinical manifestations in cattle (e.g., enteritis, pneumonia, abortion), YPS has rarely been reported in association with mastitis. Though, YPS has been identified in bovine mastitis milk (18), in mastitis samples of dairy cows (19), as well as in subclinical mastitis cases (20, 21). Thus, a characterisation of isolates can contribute to a better evaluation of phylo−/virulotypes associated with mastitis.

According to their biochemical typing profiles, YPS can be distinguished into four biotypes (BTs), of which BT1 and BT2 are highly prevalent (22). Serotyping further enables the prediction of 21 O-types (23). O:1a strains are in particular classified as important gastroenteric pathogens in Europe and have been isolated from human and non-human sources worldwide. However, a prediction of the YPS virulence is challenging as determinants are numerous and encoded by both chromosomal and extrachromosomal sequences. Main virulence determinants have been reported to be associated with an increased YPS virulence, such as the presence of the *Yersinia* virulence plasmid pYV, the chromosomally located pathogenicity islands HPI (high-pathogenicity island) and YAPI (*Yersinia* adhesion pathogenicity island), and the *Yersinia*-derived mitogen (YPM) superantigen (24). The ~70 kb plasmid pYV encodes a type III secretion system and effector proteins, some of which are toxins (25–27). HPI encodes components necessary for iron uptake and transport, including the synthesis of the siderophore yersiniabactin (28). YAPI contains genes that enable YPS to adhere to host cells as well as genes for a type IV pilus structure associated with pathogenicity and general metabolic functions.

As most of these main virulence determinants have been found to be mobile, their presence or absence appear to be the result of evolutionary adaptations of YPS to prevailing conditions (25, 29, 30).

To improve the understanding of the epidemiology of diseases caused by YPS in different animals, detailed information regarding the genomic features of YPS isolates was determined. Apart from classical typing, this study uses a genome-based approach to provide deeper insights into the virulome and the phylogenetic relationship of YPS isolates associated with a mastitis case in a cow in Germany.

## 2 Materials and methods

### 2.1 Sampling and recovery of *Yersinia pseudotuberculosis*

The YPS isolates originate from a cow after calving from a milk-producing company in Germany. The farm with 128 German “Simmentaler Fleckvieh” cows, was designated to be well-managed with a herd yield in 2025 of 11,453 kg milk with 3.98% fat and 3.62% protein. The farm facilitates open barns with straw-bedded deep stalls and a milking parlour. The udder health indicators at the farm were assessed to be very good by routine investigations in recent years. Based on milk performance monitoring, according to ICAR guidelines (International Committee for Animal Recording; https://www.icar.org/guidelines/), 74% of dairy cows had a somatic cell count (SCC) below 100,000 cells/ml, 4% above 400,000 cells/ml, and the new infection rate was 15% in 2025.

Based on the guideline of the German Veterinary Medical Society from 2018 (“laboratory diagnostics of mastitis—sampling and microbiological examination”) (31), single animal quarter milk samples were taken after cleaning and disinfection of the teat in sterile milk specimen tubes with lyophilised bacteriostat (Nerbe plus, Winsen, Germany). Milk samples were smeared on tryptic soy agar (TSA) with 5% sheep blood (BD, Becton Dickinson, Germany) and Gassner agar (Merck, Darmstadt, Germany) and were incubated aerobically for 24 to 48 h at 37 °C. In case of *Yersinia* sp. detection, the growth of small grey and yellow colonies was observed on TSA and Gassner agar, respectively. These colonies were analysed using MALDI-TOF MS (matrix-assisted laser desorption/ionisation time-of-flight mass spectrometry, MALDI Biotyper^®^ sirius system) by a direct transfer of bacterial cells and subsequent application of HCCA matrix as recommended by the manufacturer (Bruker Daltonics GmbH, Bremen, Germany). Quality scores of >2.0 were used for initial species assignment (6).

### 2.2 Bacterial cultivation and typing

*Yersinia pseudotuberculosis* isolates CVUAS 39826 (25-YE00352, sampling date: 2025-09-23), A25184579 (25-YE00385, sampling date: 2025-09-16) and A25195961 (25-YE00386, sampling date: 2025-10-02) were sent by the Chemical and Veterinary Analysis Agency (CVUA) Stuttgart to the “Consiliary Laboratory for *Yersinia* in Food” (KL-Yersinia) hosted by the German Federal Institute for Risk Assessment (BfR) (Table 1). Unless otherwise indicated, cultivation was conducted under aerobic conditions at 28 °C for 18–24 h using lysogeny broth (LB)-based media. For solid media preparation, LB medium was supplemented with 1.8% bacto agar no. 1 (Oxoid Deutschland GmbH, Wesel, Germany) (32).

**Table 1:** YPS isolates used in this study.

YPS BfR-ID [CVUA ID]	Source	Origin	Sampling date	Biotype (BT)	CR-MOX agar (pYV)	Reference
25-YE00352 [CVUAS 39826]	Cow	Stable A, Germany	2025-09-23	BT1	–	This study
25-YE00385 [A25184579]	Cow	Stable A, Germany	2025-09-16	BT1	–	This study
25-YE00386 [A25195961]	Cow	Stable A, Germany	2025-10-02	BT1	–	This study

At the KL-Yersinia, initial cultivation of YPS was performed on Columbia agar supplemented with 5% sheep blood (CSA; bioMérieux Deutschland GmbH, Nürtingen, Germany) at 28 °C for 18 h for whole-cell matrix-assisted laser-desorption/ionisation time-of-flight mass spectrometry (MALDI-TOF MS). According to the recommendation of Bruker Daltonics GmbH & Co. KG (Bremen, Germany), the direct transfer method with HCCA matrix was applied for Biotyper identification. MALDI-TOF MS scores of >2.3 were used for species prediction (5).

Initial growth of YPS was determined on *Yersinia* CIN (cefsulodin-irgasan-novobiocin) agar at 28 °C for 20–48 h. Growth on Congo red magnesium oxalate (CR-MOX) agar was used for the identification of the *Yersinia* virulence plasmid (pYV) after incubation at 37 °C for 20–24 h (33). Biochemical differentiation by biotyping was conducted as previously described. The YPS biotype was determined by metabolization of melibiose and raffinose, as well as the metabolic conversion of citrate. In addition, lipase production and bacterial motility at 28 and 37 °C after 24 to 48 h were examined (22).

### 2.3 Antimicrobial susceptibility testing

Determination of antimicrobial susceptibility was conducted by agar disk diffusion using cation-adjusted Mueller-Hinton (MH) plates according to the standards of the Clinical and Laboratory Standards Institute (CLSI VET03) (CLSI2020a). Assays were performed at 28 ± 2 °C for 24–28 h against discs with the specified substance concentrations (Thermo Fischer, East Grinstead, West Sussex, United Kingdom): amikacin (AMK, 30 μg), amoxicillin/clavulanic acid (AMC, 30 μg), ampicillin (AMP, 10 μg), ceftazidime (CAZ, 30 μg), chloramphenicol (CHL, 30 μg), ciprofloxacin (CIP, 5 μg), gentamicin (CN, 10 μg), cefotaxime (CTX, 30 μg), erythromycin (E, 15 μg), enrofloxacin (ENR, 5 μg), cefepime (FEP, 30 μg), florfenicol (FFC, 30 μg), imipenem (IMI, 10 μg), meropenem (MER, 10 μg), nalidixic acid (NAL, 30 μg), norfloxacin (NOR, 10 μg), oxolinic acid (OA, 2 μg), oxytetracycline (OT, 30 μg), streptomycin (S, 10 μg), trimethoprim/sulfamethoxazole (SXT, 1.25/23.75 μg), tetracycline (TE, 30 μg), trimethoprim (W, 5 μg) and cephalexin (CL, 30 μg). The *Escherichia coli* ATCC 25922 strain was used as quality control for AST assays (6).

### 2.4 Genomic macrorestriction and plasmid profiling

To determine the genomic diversity of the YPS isolates, pulsed-field gel electrophoresis (PFGE) was performed according to the PulseNet protocol^1^ (34). Restriction profiles were generated using NotI endonuclease (Thermo Scientific, Schwerte, Germany; treatment: 4 h at 37 °C) and separated by an initial switch time of 2.2–54.2 s for 20 h with an included angle of 120 and 6 V/cm. For plasmid prediction, agarose plugs were treated with S1-nuclease (Takara Bio Europe, Saint-Germain-en-Laye, France; 5 U) for 45 min at 37 °C, followed by addition of 10 mM EDTA and an incubation period of 15 min at room temperature to inactivate the enzyme. Electrophoresis was performed on a CHEF-DR III system (Bio-Rad, Feldkirchen, Germany) using pulsing times of 1–25 s, a total run time of 18 h, a voltage of 6 V/cm and an included angle of 120 V. After GelRed staining (concentration: 10.000 x in H2O; Genaxxon bioscience, Ulm, Germany) and destaining in water for 30 min each, the results were documented. Band patterns were evaluated in comparison to a XbaI endonuclease-treated *Salmonella* Braenderup strain H9812 genome standard using Bionumerics (v 7.6.3; Applied Maths, Ghent, Netherlands) (5, 34).

### 2.5 Illumina whole-genome sequencing and bioinformatic analysis

Genomic DNA was prepared from YPS 25-YE00352 suspensions cultivated at 28 °C for 24 h using the PureLink genomic DNA preparation mini kit (Thermo Fisher Scientific, Hennigsdorf, Germany). DNA libraries for short-read sequencing were prepared according to the standard protocol and subjected to WGS on an Illumina NextSeq500 benchtop device. Paired-end sequencing was performed in 1×149 cycles using the Illumina NextSeq Mid Output Kit (v2.5, 300 cycles; Illumina, San Diego, CA, United States) (35). Raw reads were subjected to quality evaluation using the in-house Aquamis pipeline^2^ (v1.4.1) using fastp (read trimming/read quality check (QC)), shovill (de-novo assembly), mash (reference search/species determination), QUAST v5 (assembly QC), confindr (inter/intra genus contamination analysis) and kraken2 (read/assembly based taxonomic profiling). In-silico typing of the genome sequences was conducted using the in-house Bakcharak pipeline (v3.1.6) including detection of antimicrobial resistance genes (AMRfinder [v3.12.8], NCBI resistance gene database [v2024-01-31.1]), plasmids (Abricate [v1.0.1], plasmidfinder [v2.0.1], database [v2022-07-13]), virulence factors (Abricate [v1.0.1], VFDB (yersinia) [v2024-06-25]), best genome references (mash [v2.3], NCBI refseq [October 2025]) and plasmid references (mash [v2.3], NCBI plasmid database [v2.14.1]) including PlasmidBlaster (Blastn [v2.14.1], NCBI plasmid database [October 2025]), plasmid prediction (platon [v1.6], platon database [v1.5.0, 10.5281/zenodo.4066768]) and (fastANI [v1.33], NCBI Yersinia refseq database [v2021-11-19]) are carried out. In-silico serotyping was conducted by comparative analysis at nucleotide level between the conserved gene regions of the 15 notified YPS O-antigens (serotypes O:1 to O:15) including their ten subtypes (O:1a/b/c, O:2a/b/c, O:4a/b and O:5a/b) as previously described (36). The 25-YE00352 genome was annotated using the Prokaryotic Genome Annotation Pipeline (v4.11, https://www.ncbi.nlm.nih.gov/genome/annotation_prok/). Phylogenetic comparison of genome sequences was conducted using CSI-Phylogeny (v1.4) using default parameter settings. Resulting Newick files were implemented into Figtree for graphical illustration. Genomes were derived out of public database entries as well as from our internal collection as specified.

### 2.6 Nucleotide sequences availability

The nucleotide sequence of 23-YE00352 (CVUAS 39826) is available in bioproject PRJNA1358262 (*Yersinia pseudotuberculosis* from a mastitis infection in Germany) under accession (JBWJNY000000000).

## 3 Results

### 3.1 *Yersinia pseudotuberculosis* caused mastitis in a dairy cow on a German farm

The cow affected by YPS-associated mastitis calved for the second time at the end of April 2025. Before calving SCC was below 100,000 cells/ml, while determination after calving showed an increased SCC above 180,000 cells/ml. Clinically, the cow was unremarkable (no flaky milk). In three subsequent samplings in September (*n* = 2) and October (*n* = 1), YPS was detected (Table 1). Except for milk of one quarter, no YPS could be found, also not in the faeces of the cow. As YPS was detected, the cow was no longer milked on the affected quarter. Before drying off, microbiological re-investigation indicated that still low numbers of YPS were detectable. The cow was treated with cefalonium for drying off and tested again after calving. Thereafter, YPS could not be detected anymore, but the SCC remained increased at the affected quarter with 984,000 cells/ml. During herd samplings in October 2025 and January 2026, all cows were investigated using the California Mastitis Test and samples were taken from all positive quarters for bacteriological investigation. Only unspecific growth or the occurrence of coagulase-negative staphylococci, aesculin-positive streptococci or other coliforms were noted.

At the KL-Yersinia, the recovered isolates 25-YE00352, 25-YE00385 and 25-YE00386 were confirmed as YPS by both mass spectrometry and classical biotyping focusing on Voges-Proskauer (negative), sorbitol (negative), raffinose (positive/negative), sucrose (negative) and melibiose (positive/negative) reactions. Biotype-associated reactions of citrate, melibiose and raffinose lead to an assignment to biotype BT1 for all YPS. The biochemical reactions are shown in Table 2 (Supplementary Table S1).

**Table 2:** Growth of YPS and biochemical typing results.

Characteristic	25-YE00352
Growth	
CIN (*Yersinia*-like)^a^	+
CR-MOX (pYV)^b^	−
Motility	
SIMA 25 °C (37 °C)	− (−)
Biochemical reaction	
Lipase	+
Urease	+
Citrat	−
Mukat	−
Ornithin	−
VP 25 °C (37 °C)	− (−)
Sugar utilisation	
Aesculin	+
Cellobiose	−
Indol	−
Melibiose	+
Rhamnose	+
Saccharose	−
Salicin	−
Sorbose	−
Trehalose	+
Xylose	+
Raffinose	−
Species (biotype)	**YPS (BT1)**

a YPS colonies: deep-red centre with a translucent, sharp border (bull’s-eye).

b pYV-positive: small orange-red colonies, pYV-negative: large colourless colonies.

CIN, cefsulodin-irgasan-novobiocin; CR-MOX, Congo red magnesium oxalate; SIMA, Simmons citrate agar; VP, Voges Proskauer; BT1, biotype 1.

Antimicrobial susceptibility testing against 24 substances of 11 different antibiotic classes revealed that all YPS exhibited wildtype phenotypes (susceptible) for almost all tested substances including cephalosporins of the 1st generation (i.e., cephalexin) used for treatment of the infection in the cow. Except erythromycin, large inhibition zones as specified in Table 3 (Supplementary Table S2) were observed for the remaining antibiotics during AST testing. However, erythromycin resistance is very common in YPS and is referred as an intrinsic resistance.

**Table 3:** Results of agar disc diffusion for YPS 25-YE00352.

Antibiotic substance (abbreviation)	Inhibition zone (mm)
Cefotaxime (CTX)	38
Streptomycin (S)	18
Tetracycline (TE)	25
Imipenem (IMI)	31
Amikacin (AMK)	23
Chloramphenicol (CHL)	24
Gentamicin (CN)	19
Norfloxacin (NOR)	27
Meropenem (MER)	34
Amoxicillin/clavulanic acid (AMC)	31
Nalidixic acid (NAL)	28
Erythromycin (E)	6
Trimethoprim (W)	26
Trimethoprim/sulfamethoxazole (SXT)	23
Ampicillin (AMP)	31
Ciprofloxacin (CIP)	31
Florfenicol (FFC)	26
Ceftazidime (CAZ30)	32
Cefepime (FEP)	35
Enrofloxacin (ENR)	32
Oxytetracycline (OT)	25
Oxolonic acid (OA)	29
Cephalexin (CL)	27
Ceftazidime (CAZ10)	31

mm, millimetre.

### 3.2 *Yersinia pseudotuberculosis* strain 25-YE00352 belongs to bioserotype 1/O:2b ST16 and is related to isolates from zoo animals

**Figure 1:**
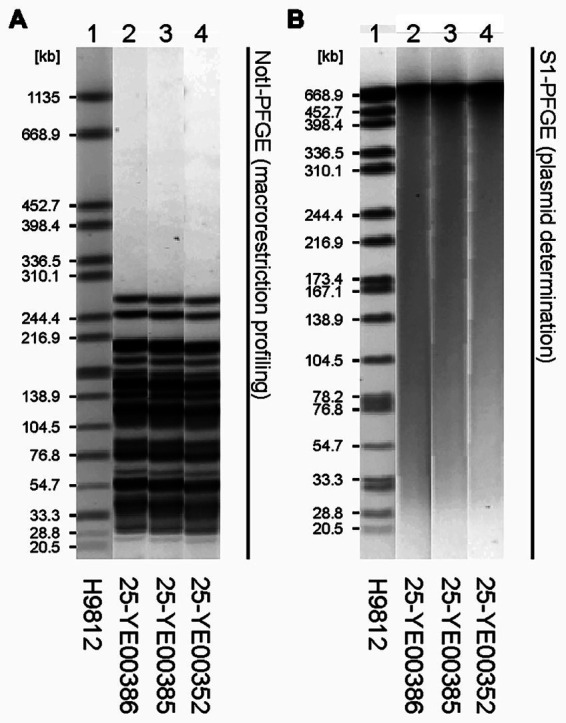
Macrorestriction profiling and plasmid prediction of the YPS isolates. **(A)** The patterns of the genomic DNAs after treatment with restriction endonuclease NotI are shown. **(B)** Plasmid detection was conducted using S1 nuclease-treatment of the genomic DNAs. The lanes indicate the isolates as indicated below the photographs. As reference, the *Salmonella* serovar Braenderup strain H9812 restricted with XbaI was used.

Macrorestriction profiling revealed an identical NotI-pattern for all YPS isolates suggesting a repeated isolation of the same strain (Figure 1A). In addition, none of the isolates provided hints for the presence of extrachromosomal elements (plasmids), neither the common 70 kb virulence plasmid (pYV), nor other plasmids (Table 2; Figure 1B). For in-depth characterisation, isolate 23-YE00352 was selected and subjected to WGS and bioinformatics analysis. The mass spectrometric species assignment was confirmed by an appropriate average nucleotide identity (ANI) value of 99.3% of the 25-YE00352 genome against the YPS IP32953 reference sequence (GCF_000834295.1) (Supplementary Table S3). In-silico prediction of acquired AMR and chromosomal alteration of genes associated with resistance development did not yield matches to reference sequences backed in the AMRfinder database. In addition, the genomes did not provide any indication of further determinants associated with resistances against biocides, heat, acid or heavy metals (Supplementary Table S3).

**Figure 2:**
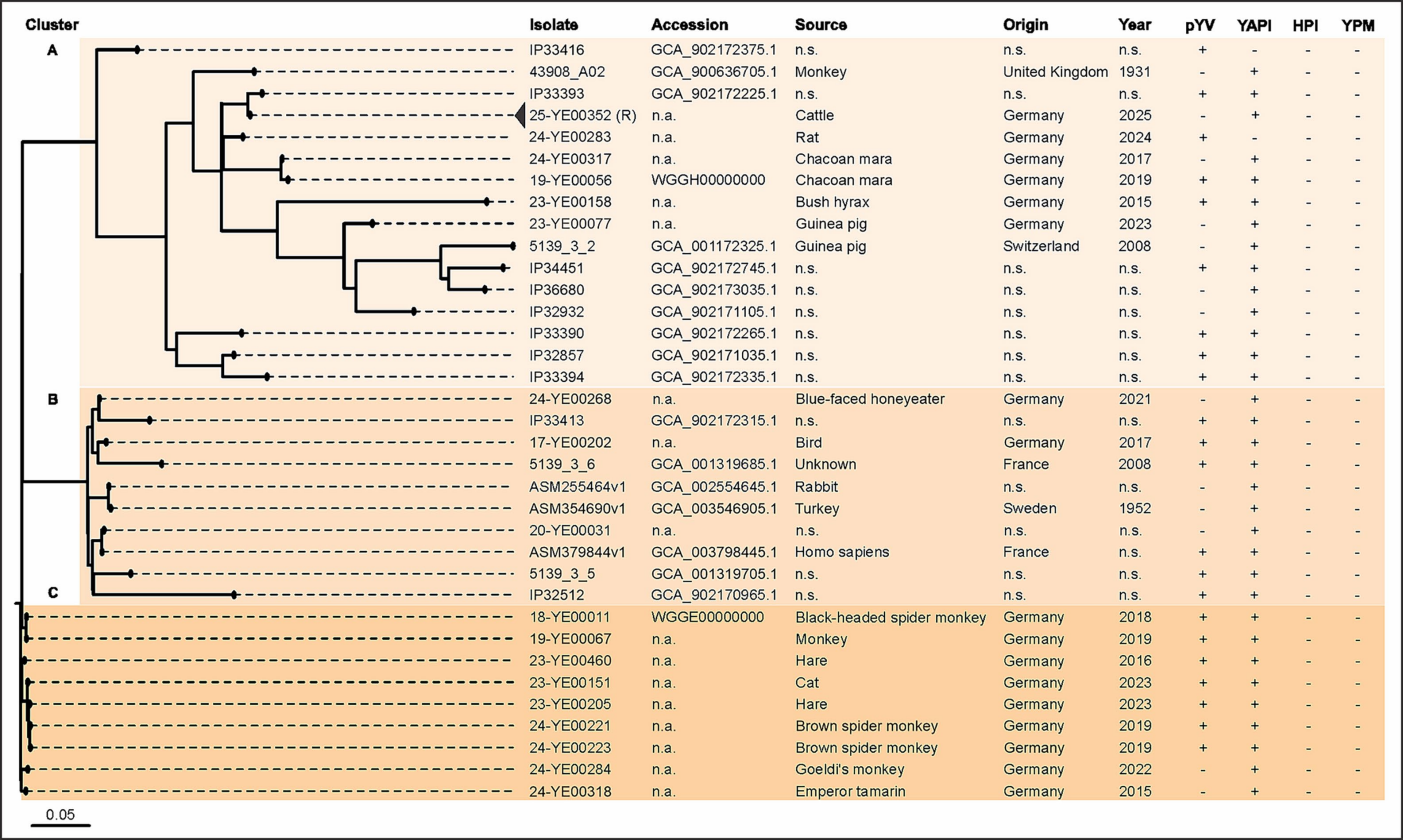
Phylogenetic relationship of 25-YE00352 to other YPS genomes of sequence type ST16. CSI-Phylogeny (v1.4) was used for the generation of a SNP-tree against the 25-YE00352 genome as a reference (default parameter settings). 3,852,970 of 4,773,355 nucleotide positions of the reference genome (80.72%) were found to be conserved in all analysed genomes. The ruler indicates the number of substitutes per sites of the phylogenetic tree. On the right, available metadata were added. n.a., not available; n.s., not specified; pYV, plasmid *Yersinia* virulence; YAPI, *Yersinia* adhesion pathogenicity island; HPI, high pathogenicity island; YPM, *Yersinia pseudotuberculosis* mitogen.

According to the composition of genes associated with the O-antigen cluster as previously described (36), the isolate was assigned to the serotype O:2b. Based on the allele profile of the housekeeping genes (*adk*-4, *argA*-2, *aroA*-10, *glnA*-2, *thrA*-2, *tmk*-5, *trpE*-1) specified in the pubMLST Achtmann scheme-3, the genome of YPS 25-YE00352 belongs to sequence type ST16 (Supplementary Table S3). In a broader context, 25-YE00352 phylogenetically closely resembles ST16 genomes esp. from Germany in comparison to all globally available ST16 genomes (*n* = 34; Figure 2) (Supplementary Table S4). In general, the cgMLST-based tree led to a separation of three distinct clusters (A, B, and C) further subdivided into certain subclusters. Closely related genomes are notified to be originating from animals, especially various zoo animal orders (Figure 2).

### 3.3 25-YE00352 contains YAPI, but lacks other main virulence determinants including pYV, YPM, and HPI

Overall, the genome of 25-YE00352 exhibited a total of 161 virulence-associated genes of entries in the virulence factor database for Yersinia (VFDB). Functionally, the gene products contribute to adherence (*n* = 18), effector delivery systems (*n* = 42), exoenzymes (*n* = 1), exotoxins (*n* = 2), immune modulation (*n* = 14), invasion (*n* = 4), motility (*n* = 49) as well as nutritional/metabolic (*n* = 12) and others pathways (*n* = 17) that may be associated with virulence (Supplementary Table S3). Due to the impact of specific factors for virulence, 25-YE00352 was further analysed for the presence of pYV, HPI, YAPI and the YPS-derived mitogen. Initial cultivation of the isolate on CR-MOX agar resulted in large colourless colonies indicating an absence of pYV (Table 2; Supplementary Table S1). The phenotypic result is in agreement with the S1-PFGE plasmid prediction as well as with the in-silico analysis, as none of the components of pYV-associated virulence factors could be determined in the WGS dataset. On the contrary, the 25-YE00352 genome exhibited the *pil* operon (*pilLMNOPQRSUV*), encoding a type IV pilus that contributes to pathogenicity that is associated with YAPI. Factors encoded by HPI as well as the YPS-derived mitogen were not identified in 25-YE00352.

## 4 Discussion

The zoonotic pathogen YPS poses a serious threat to animal health, although it affects humans to a lesser extent. Infection in animals often occurs unrecognised as the symptoms of the disease are rather unspecific, but frequently resulting in fatal diseases (6, 15). The broad distribution of YPS in the environment, as well as in domestic and wild animals, challenges the identification of sources and chains of infection. YPS is generally considered to be pathogenic, but the presence of different main virulence determinants (i.e., pYV, HPI, YAPI and YPM) has led to the definition of distinct virulotypes associated with increased virulence (24). However, isolates lacking all of these determinants were also found in fatal infection (25–27). Thus, these factors may be less important for a general infection of animals, but probably affect the host specificity or the manifestation of disease (fatal vs. chronic). The high diversity of observed virulotypes, together with the constitution of the animal and environmental stresses make it difficult to evaluate the impact of specific isolates on animal health.

So far, mastitis infection in cattle caused by YPS has only rarely been reported (18–21). Data about YPS involved in infections are limited, but indicate an implication of isolates of the bioserotype 1/O:1. This type is very common in human infections in Europe, but has also been identified in human and non-human reservoirs worldwide (9). Genetically, available YPS genomes from mastitis infections belong to the phylotype ST42, a phylotype that is highly prevalent globally. ST42 genomes of public databases in general show a high diversity in their virulotypes by exhibiting the main virulence determinants in certain combinations (37). YPS isolates from mastitis infections in cows in Italy have been shown to exhibit pYV, HPI and YAPI and were thus assigned to have a high virulence (19). The isolates described here belong to bioserotype 1/O:2b and phylotype ST16. The ST16 phylotype is less prevalent amongst the globally described genomes in Enterobase, but also individual isolates may exhibit different virulotypes ranging from YPS with different combination of the primary virulence determinants (e.g., pYV, YAPI) to isolates lacking all of them (Supplementary Table S4). The close relationship of the mastitis isolates described here to genomes of YPS that caused fatal cases of captive animals (i.e., from North Rhine Wesphalia) (4) in Germany (Figure 2) may indicate an emergence of a geographic local phylotype. While 25-YE00352 carries YAPI, it lacks the remaining main virulence determinants (i.e., pYV, HPI and YPM) suggesting a moderately increased virulence in comparison to YPS lacking all these factors. Further dissection of YPS isolates associated with mastitis will provide a deeper insight on the virulome necessary for this disease. As the virulomes of the different YPS sequence types do not differ substantially, the impact of the phylotypes on host specificity, animal infection and disease development need to be determined for a reliable risk evaluation.

The absence of the virulence plasmid has been confirmed for all YPS isolates recovered during the mastitis infection period, indicating a general absence of pYV. However, some reports have shown that pYV-deficient isolates can also be involved in severe infection (24, 25). Successive subcultivation of YPS can result in the loss of pYV. However, pYV-deficient isolates can acquire the virulence plasmid from other Yersiniae through horizontal gene transfer. Some conjugative plasmids (e.g., pYE854/pYE966) have been notified to support its transmission with moderate frequencies (32, 33). However, the isolates from mastitis infection in this study do not exhibit any extrachromosomal element that may support the uptake of plasmids. Also, the pathogenicity islands have been shown to be mobile and can be thus acquired or lost as a consequence of bacterial adaption processes in response to environmental or artificial stresses (24, 30).

A treatment of the 25-YE00352-associated YPS mastitis infection seems feasible using antimicrobials, as phenotypic testing by agar disc diffusion revealed wild-type phenotypes (susceptibility) to almost all of the tested antibiotics including 1st generation cephalosporins used for treatment of the mastitis infection in the cow. In addition, no resistance determinants for antibiotics, biocides or heavy metals were found in the in-silico analysis. However, the isolate showed a high minimal inhibitory concentration against colistin (>16 mg/L, data not shown) as performed by broth microdilution according to the CLSI guidelines (data not shown). As none of the different mobile colistin resistance genes and variants were found, the phenotype needs to be mediated by one of the different chromosomal-mediated resistance mechanisms as previously described (e.g., *phoPQ*, *pmrAB*) (38). So far, antimicrobial resistance in *Yersinia* from wildlife or the environment has been reported to be generally low (5, 6, 39, 40). Nevertheless, human and livestock YPS occasionally exhibited acquired AMR determinants, albeit at a very low level (39, 40). To prevent multidrug-resistance development and spread, treatment of YPS in animals with antimicrobials should therefore be carried out carefully.

In conclusion, mastitis caused by YPS is rare, but seems to be associated with different bioserotypes and phylotypes. The ST16 YPS described here represents a less prevalent phylotype, of which a substantial number of published genomes originate from German zoo animals. The source of the mastitis infection remains unknown, but may be driven by the acquisition of YPS from the environment, from wildlife populations, or by spread of YPS through asymptomatic carriers. The study indicates that ST16 YPS can be implicated in chronic mastitis infection in cattle.

## Data Availability

Sequencing data for this study can be found at NCBI Genbank under bioproject PRJNA1358262.

## Footnotes

1
http://www.cdc.gov/pulsenet/

2
https://gitlab.com/bfr_bioinformatics/AQUAMIS

## Supplementary material

SUPPLEMENTARY TABLE S1 Routine typing results of YPS isolates.

SUPPLEMENTARY TABLE S2 Results of antimicrobial susceptibility testing of YPS isolates.

SUPPLEMENTARY TABLE S3 Summary on genome features for YPS 25-YE00352.

SUPPLEMENTARY TABLE S4 Summary on genome features for publicly available YPS ST16 genomes from Enterobase.
